# Combined ultrasound‐guided hyaluronic acid injections, high‐power laser therapy and physiotherapy improve hip function in osteoarthritis: A randomized controlled trial

**DOI:** 10.1002/jeo2.70685

**Published:** 2026-05-25

**Authors:** Alessandro de Sire, Nicola Marotta, Isabella Bartalotta, Emanuele Prestifilippo, Roberta Zito, Valerio Ammendolia, Federica Pisani, Francesco Agostini, Umile Giuseppe Longo, Antonio Ammendolia

**Affiliations:** ^1^ Department of Medical and Surgical Sciences University of Catanzaro ‘Magna Graecia’ Catanzaro Italy; ^2^ Research Center on Musculoskeletal Health, MusculoSkeletalHealth@UMG University of Catanzaro ‘Magna Graecia’ Catanzaro Italy; ^3^ Physical and Rehabilitative Medicine Division, Department of Experimental and Clinical Medicine University of Catanzaro ‘Magna Graecia’ Catanzaro Italy; ^4^ Department of Anatomical and Histological Sciences, Legal Medicine and Orthopedics Sapienza University Rome Italy; ^5^ Department of Orthopaedic and Trauma Surgery Fondazione Policlinico Universitario Campus Bio‐Medico Roma Italy; ^6^ Research Unit of Orthopaedic and Trauma Surgery, Department of Medicine and Surgery Università Campus Bio‐Medico di Roma Roma Italy

**Keywords:** functioning, hip osteoarthritis, hyaluronic acid, lasertherapy, rehabilitation

## Abstract

**Purpose:**

High‐power laser therapy (HPLT) is effective in treating musculoskeletal conditions, reducing pain and improving joint functionality. This randomized controlled trial (RCT) aimed to explore the effectiveness of a combination of HPLT, hyaluronic acid injections and rehabilitation for patients with hip osteoarthritis (OA).

**Methods:**

This RCT enroled adults over 18 with a diagnosis of hip OA (Stages 1–3) who experience hip pain of 4 or higher on a scale. Participants must have a body mass index (BMI) under 30. Patients were randomly assigned to either a study or control group, with both patients and assessors blinded to the treatment. The study group received hyaluronic acid injections, physiotherapy and active HPLT, while the control group received the same management, except for sham HPLT. We used the Harris Hip Score (HHS) as primary outcome to assess hip functioning, and as secondary outcomes the following ones: Numeric Rating Scale (NRS) for pain, EuroQol‐5 Dimension (EQ‐5D) for quality of life and the 6‐Minute Walking Test for physical performance. Evaluations were conducted at multiple time points: baseline (T0), post‐treatment (T1) and follow‐ups at 3 months (T2), 6 months (T3) and 12 months (T4) after the first visit. Moreover, 57 patients were randomly assigned to either a study group (*n* = 28) or a control group (*n* = 29).

**Results:**

Both groups showed significant initial improvements in HHS from T0 to T1 (*p* < 0.001). However, statistically significant intergroup differences favouring the study group were found for the 6‐Minute Walk Test (*p* = 0.029) and the EuroQoL at T3 (*p* = 0.030).

**Conclusion:**

This RCT demonstrated that hyaluronic acid injections and physiotherapy, combined with laser therapy, improve hip function and pain perception in hip OA patients. While laser therapy showed promise, more evidence is needed.

**Level of Evidence:**

Level I.

**Abbreviations**
6MWT: 6‐Minute Walk Test
ANOVA: analysis of variance
BMI: body mass index
EQ‐5D: EuroQol‐5 Dimension
HHS: Harris Hip Score
HPLT: high‐power laser therapy
KOA: knee osteoarthritis
MMSE: Mini‐Mental State Examination
MMT: Manual Muscle Testing
MRC: Medical Research Council
NRS: Numeric Rating Scale
NSAIDs: nonsteroidal anti‐inflammatory drugs
OA: osteoarthritis
RCT: randomized controlled trial
ROM: range of motion

## INTRODUCTION

The hip joint is one of the primary joints commonly affected by degenerative osteoarthritis (OA) [28]. Therefore, hip OA is a major cause of hip pain leading to significant mobility limitations and a worsening in physical capacity, impacting functional independence and increasing the reliance on healthcare services [13]. Moreover, this condition might severely affect daily activities such as walking, climbing stairs and rising from a chair, with significant repercussions on the patient's quality of life [41].

Hip OA is a major cause of pain, leading to significant mobility limitations, reduced physical capacity and severe impact on the patient's quality of life. Pain is the predominant symptom, often accompanied by stiffness and functional limitation [25, 37]. It is well known that joint damage compromises the integrity of the capsule, which is rich in kinaesthetic receptors, potentially affecting proprioception and postural stability [13]. However, the literature offers few studies, and those available present conflicting results regarding postural changes associated with hip joint damage [5].

The management of OA focuses on pain relief and incorporates both pharmacological and non‐pharmacological approaches, such as therapeutic and physical exercises, as well as weight loss and changes to lifestyle habits [17]. Chronic pain is the main concern for patients with OA and the primary reason they seek medical care; however, in addition to pain, patients often could experience related comorbidities, such as insomnia and depression, which can amplify their pain experience [18].

Therapeutic options for OA available include both medications and surgical interventions. Commonly used medications to alleviate symptoms include topical analgesics, nonsteroidal anti‐inflammatory drugs (NSAIDs), glucocorticoid injections directly into the joints and hyaluronic acid [20].

Ultrasound‐guided intra‐articular injection of hyaluronic acid is widely recognized as an effective therapeutic strategy for relieving pain, improving joint function and potentially slowing the progression of OA [27]. Thanks to its unique properties, hyaluronic acid is particularly suitable for improving rehabilitative management. Its high viscosity reduces friction between joint surfaces, facilitating and promoting movement, while its hydrophilic properties allow it to absorb water and maintain joint hydration, which is essential for optimal joint function [12, 21].

Various formulations of hyaluronic acid are available for the clinical management of OA, including linear and cross‐linked formulations. It is worth noting that the natural hyaluronic acid present in healthy synovial fluid is of the linear type, whereas the cross‐linked formulation allows for the formation of compounds with a higher molecular weight, which is then used for therapeutic purposes [14, 38].

Emerging literature suggests that integrating intra‐articular therapies with rehabilitation interventions may enhance clinical outcomes in patients with OA, supporting a multimodal and patient‐centred approach [44].

In clinical practice, therapeutic exercises and hyaluronic acid injections are commonly used strategies, but their synergistic potential has not yet been fully explored [13, 14, 15, 16].

In particular, it has been recently demonstrated that the key role of ultrasounds in guiding the intra‐articular injections, particularly in patients affected by knee OA [7, 32].

In the context of rehabilitation, the best results are achieved when healthcare professionals adopt a multimodal approach, integrating various therapies and when patients actively participate in managing their pain [2, 24]. Among the physical agent modalities commonly used in rehabilitation, laser therapy is a non‐surgical treatment modality aimed at promoting the regeneration of cartilage tissue; in fact, laser therapy operates by delivering focused light energy to tissues, stimulating cellular repair mechanisms, reducing inflammation and enhancing blood circulation in the affected area [3]. In particular, high‐power laser therapy (HPLT), due to its ability to penetrate deeper tissues, allowing targeted therapeutic effects, has been effective in managing musculoskeletal conditions [23]. Studies have demonstrated that HPLT is effective in reducing pain, improving joint function and enhancing mobility, especially when used in concurrence with physical therapy [4, 19].

Very few studies have evaluated the application of these techniques individually in the treatment of OA [6, 8, 10, 11, 22, 47], but, to date, no research has ever investigated the role of HPLT and, in particular, their combined use in hip OA patients. Therefore, the present randomized controlled trial (RCT) was designed to answer the following research question, formulated using the PICOT framework: P (Population): In adult patients with hip OA (Stages 1–3); I (Intervention): Does the combined therapeutic approach (ultrasound‐guided hyaluronic acid injections, active HPLT and physiotherapy); C (Comparison): Compared to the standard approach (ultrasound‐guided hyaluronic acid injections, sham HPLT and physiotherapy); O (Outcome): Lead to better functioning (Harris Hip Score [HHS]) and secondary outcomes (Numeric Rating Scale [NRS], 6‐Minute Walk Test [6MWT], EuroQoL); T (Time): Over a 12‐month follow‐up period (T1, T2, T3, T4).

Therefore, to address this gap, the present RCT aimed to evaluate the efficacy of a combination of ultrasound‐guided intra‐articular hyaluronic acid injections, laser therapy and rehabilitative treatment on functioning in patients with hip OA.

## MATERIALS AND METHODS

### Study participants

This RCT was conducted at the Physical Medicine and Rehabilitation Unit of the ‘Renato Dulbecco’ University Hospital. Participants were recruited at our health centre and underwent initial clinical and functional assessment, followed by the prescribed rehabilitation programme and scheduled outpatient follow‐ups. All included patients provided written informed consent for the processing of personal data and were informed of their rights. This study was approved by the Regional Ethical Committee (approval number 429/2021; date of approval: 16 December 2021) and was conducted in accordance with CONSORT guidelines, the Declaration of Helsinki and relevant regulatory requirements. The paper has been registered in the International Public Trials Registry ClinicalTrials.gov (Identifier: NCT07108400). Patients with coxarthrosis were consecutively screened for eligibility among those referred to our Physical Medicine and Rehabilitation Unit between January 2022 and September 2024 at the Complex Operating Unit of Physical Medicine and Rehabilitation of the ‘Renato Dulbecco’ University Hospital. The inclusion criteria were as follows: Age over 18 years; diagnosis of hip OA (stage ≤ 3 according to the Kellgren and Lawrence [KL] classification); hip pain assessed with the NRS ≥ 4; body mass index (BMI) less than 30 kg/m^2^; we excluded patients with KL Grade 4 OA because the study focused on conservative management, while advanced OA typically requires surgical treatment. Patients with BMI > 30 were excluded to avoid confounding factors related to excessive mechanical load and reduced rehabilitation adherence; suspension of anti‐inflammatory drugs (NSAIDs), opioids, corticosteroids, muscle relaxants or any other therapy that could interfere with the study assessments. Use of analgesic or anti‐inflammatory drugs was not permitted during the treatment period. Compliance was ensured through patient diaries and verification at each follow‐up visit.

The exclusion criteria were: Mini‐Mental State Examination (MMSE) score < 24, concurrent treatment with anti‐inflammatory drugs or rehabilitation therapies and rheumatoid arthritis.

Presence of hip prosthesis; planned lower limb surgery within 6 months; intra‐articular injections, physical therapies or physical agent modalities in the previous 30 days; history of neurological or psychiatric disorders; haemorrhagic diathesis; pacemaker wearers; oncological pathologies; pregnancy or breastfeeding; epilepsy; and current infectious diseases. Patients were thoroughly informed, both orally and in writing about their rights. After reading the information, they provided written informed consent for the processing of their personal data.

### Intervention

The enroled patients were randomly divided (using a 1:1 stratified sampling method) into two groups, a study and a control group. Both patients and the physician responsible for the outcome assessments were blinded to the group assignment. The total duration of the therapy was 2 weeks.

− Study Group: Received two ultrasound‐guided intra‐articular injections of Hymovis hyaluronic acid (24 mg/3 mL, manufactured by Fidia Farmaceutici S.P.A.) on a weekly basis, 10 sessions (5 sessions/week) of active HPLT using a diode laser and 10 sessions (5 sessions/week) of physical therapy specifically targeting hip OA, each lasting 45 min.
− Control Group: Received the same protocol, two weekly ultrasound‐guided hyaluronic acid injections and 10 sessions of physiotherapy but received 10 sessions of ‘sham’ HPLT. The sham device was used to maintain patient blinding due to the presence of a light pointer and an acoustic signal that made it impossible to distinguish the active from the simulated treatment.

All sessions, including injections, physical therapy and laser therapy with a high‐power diode laser (Laserix Pro: wavelength: 905 ± 10 nm, pulsed emission: 1000–80,000 Hz with supermodulation 10%–100% duty cycle, average power: up to 5000 mJ/s, peak power with hardware modulation of the laser source: from 600 to 1200 W and diode‐fibre‐lens handpiece), were scheduled in the morning, between 9:00 and 13:00 for both groups. Following the infiltration cycle, laser therapy and physiotherapy sessions were scheduled, and patients began treatment approximately 2–3 days later. During each treatment session, patients received both laser therapy and physiotherapy, with physiotherapy typically starting about 15 min after the laser therapy.

#### Hyaluronic acid injection

**Figure 1:**
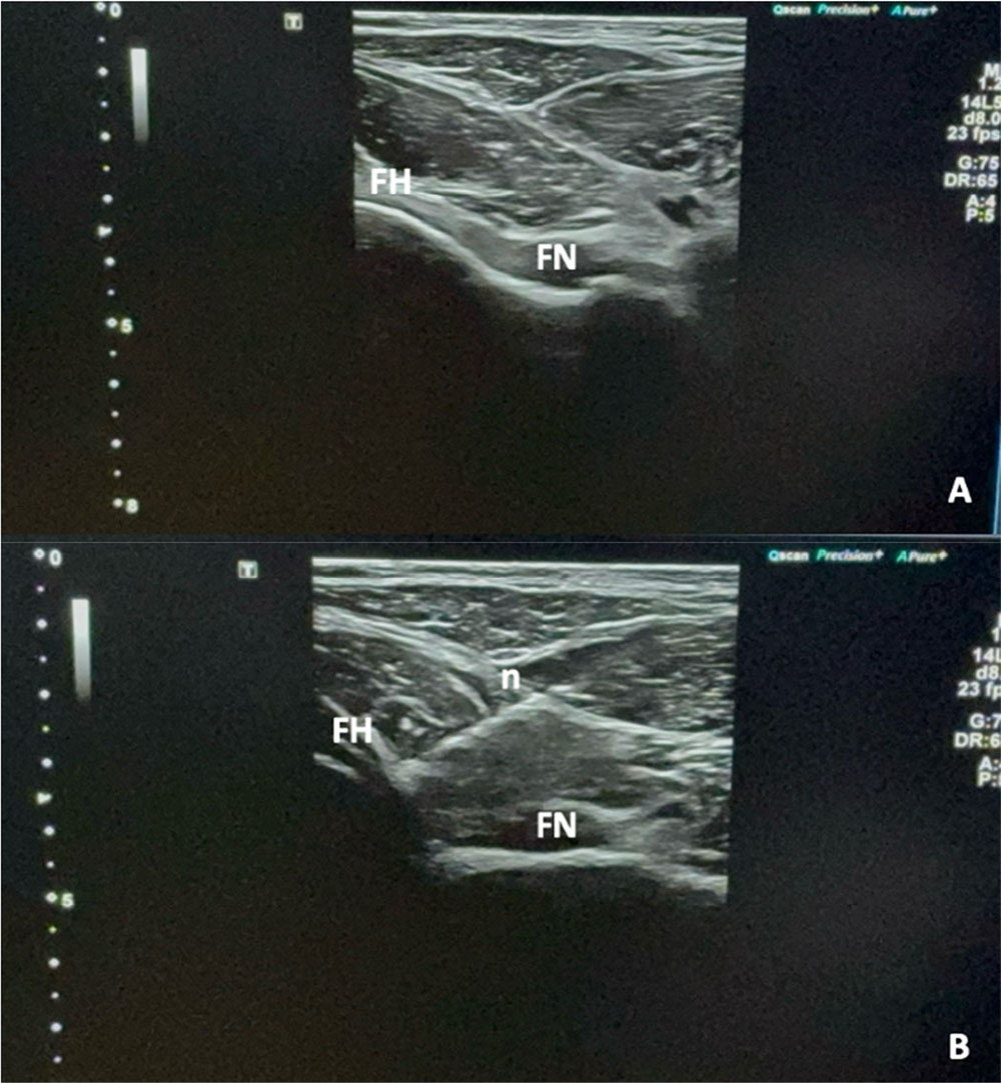
(a) Before Hyaluronic acid injection. (b) During hyaluronic acid injection. FH, femoral head; FN, femoral neck; n, needle.

The intra‐articular injection of hyaluronic acid was performed using an ultrasound guide (18–6 MHz linear array transducer Aplio A‐Canon Medical Systems Europe) to precisely target the injection site. The patient was positioned supine with the affected hip slightly medially rotated (about 15°). The injection was performed via an anterolateral approach using a 20‐gauge needle. Under real‐time ultrasound guidance, the needle was inserted and directed to the joint capsule, ensuring accuracy and safety. Colour Doppler ultrasound was performed to identify and avoid large blood vessels. All necessary precautions were taken to ensure complete sterility, including sterilization of the transducer, use of sterile gel and disinfection of the patient's skin with 10% Betadine. The hyaluronic acid was then injected precisely into the joint capsule (see Figure 1 for further details).

#### Lasertherapy

**Figure 2:**
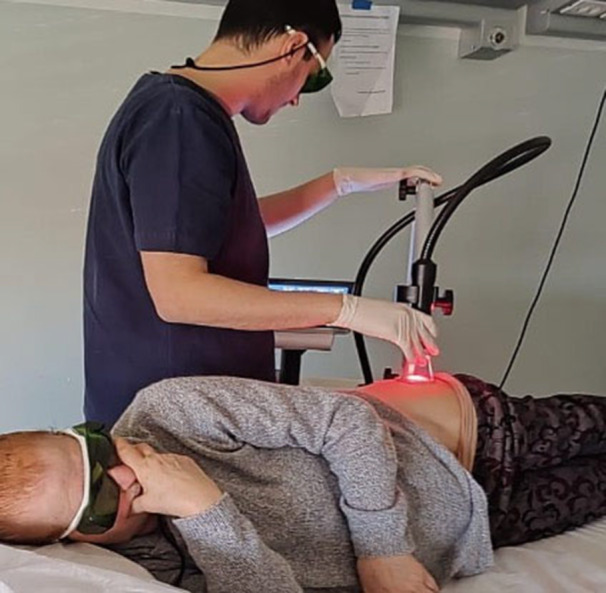
Execution of laser therapy.

After the injections of hyaluronic acid, the patient underwent laser therapy administered by an expert physiotherapist. The parameters used for the treatment with high‐power diode laser are as follows: Total energy: 2736 joules; peak power: 1200 watts; frequency: 30 kHz; duty cycle: 70%; duration: 20 min. After identifying the treatment site, the area was thoroughly disinfected with a specialized solution to eliminate any exogenous chromophores. The anterolateral access route was chosen for the laser therapy, inasmuch as this area lies between the femoral vascular‐nervous bundle and the outer margin of the acetabulum. For hip administration, a 50 mm head was used. Moreover, the handpiece was positioned at a 90° angle to the skin to minimize radiation scattering. Both the patient and the operator wore protective glasses throughout the procedure (see Figure 2 for further details).

#### Physiotherapy

**Figure 3:**
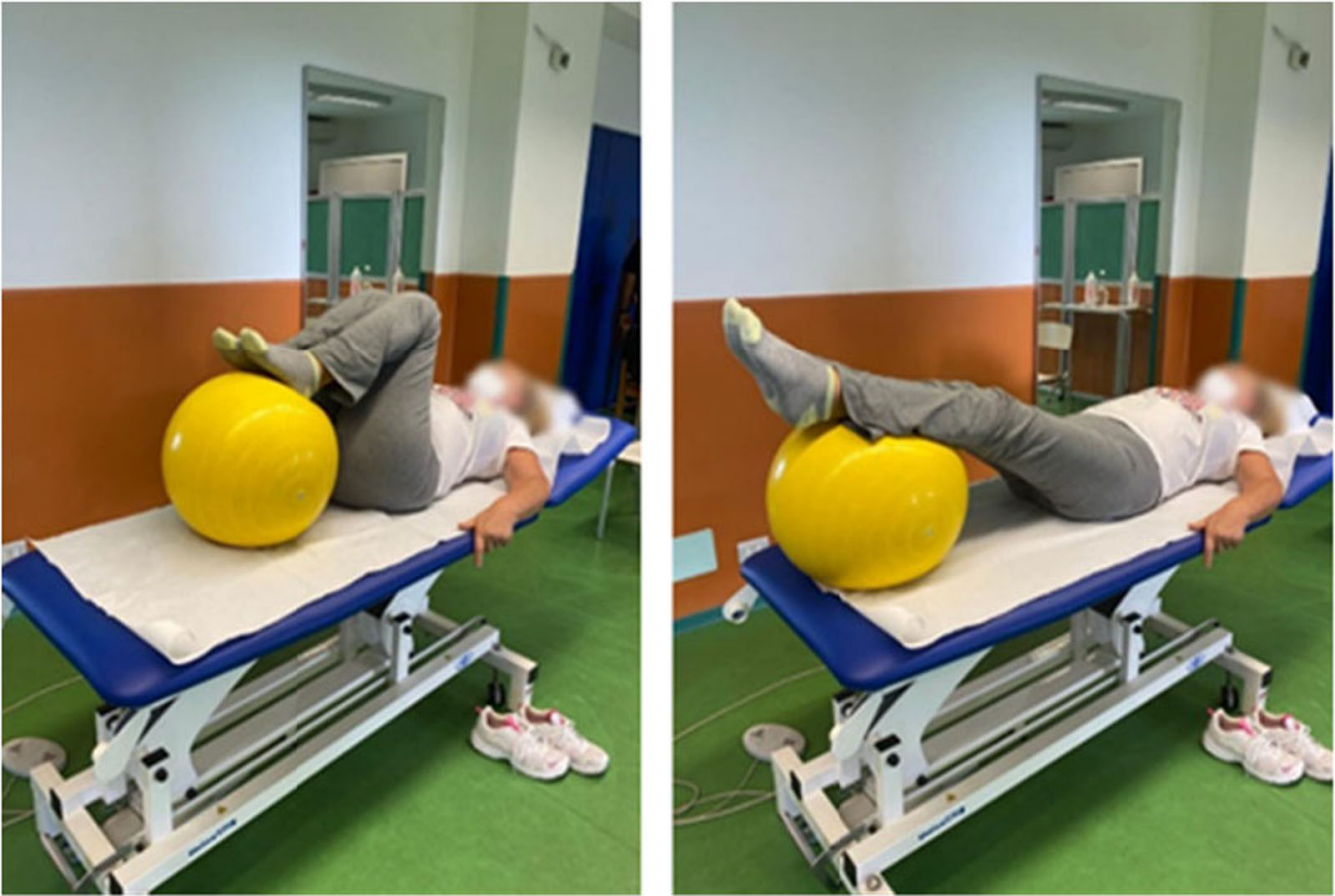
Flexion/extension with Bobath ball.

At the end of the laser therapy session, the patient shifted to a specific physiotherapy programme designed to treat hip OA. The study participants underwent 10 sessions of physical therapy specifically targeting hip OA (5 sessions/week for 2 weeks) lasting 45 min. Each rehabilitation session started with pelvic girdle mobilization, a process carefully structured into three progressive phases. The first phase, known as the guidance and perception phase, involved the physiotherapist actively guiding the patient's movements. During this stage, the patient remained passive, focusing on becoming aware of the motions being performed. Once the patient has developed a sense of the movement, the session moves into the verification phase. Here, the patient initiated engagement of muscle activation, performing controlled movements under the physiotherapist's close supervision. In the final phase, called the recruitment and strengthening phase, the patient could perform the movements while the physiotherapist applies mild manual resistance, encouraging muscle engagement and increasing strength. Another technique for mobilizing the pelvic girdle involves having the patient lie on their back in a supine position while using a Bobath ball (fitball) (see Figure 3).

**Figure 4:**
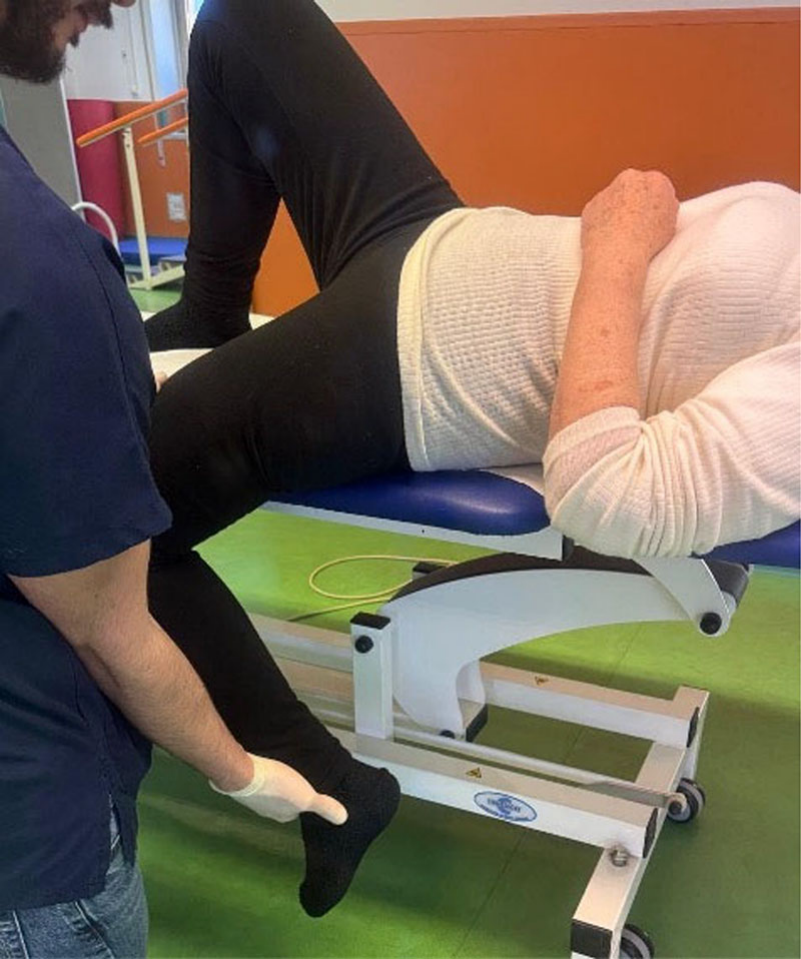
Kabat method exercises.

This approach added an element of stability training, improving joint mobility in a dynamic and controlled manner. The rehabilitation process began with passive mobilization of the lower limb, ensuring all hip movements are performed within a pain‐free range. Active physical therapy required the patient's muscular activation to improve active range of motion (ROM) and enhance muscle strength. Starting from a supine position, closed and open kinetic chain exercises are performed. Then, the rehabilitation continues with the application of diagonals following the Kabat method. After the Kabat method treatment, the patient undergoes proprioceptive and gait training, which includes exercises to improve balance using a platform with parallel bars (see Figure 4).

**Figure 5:**
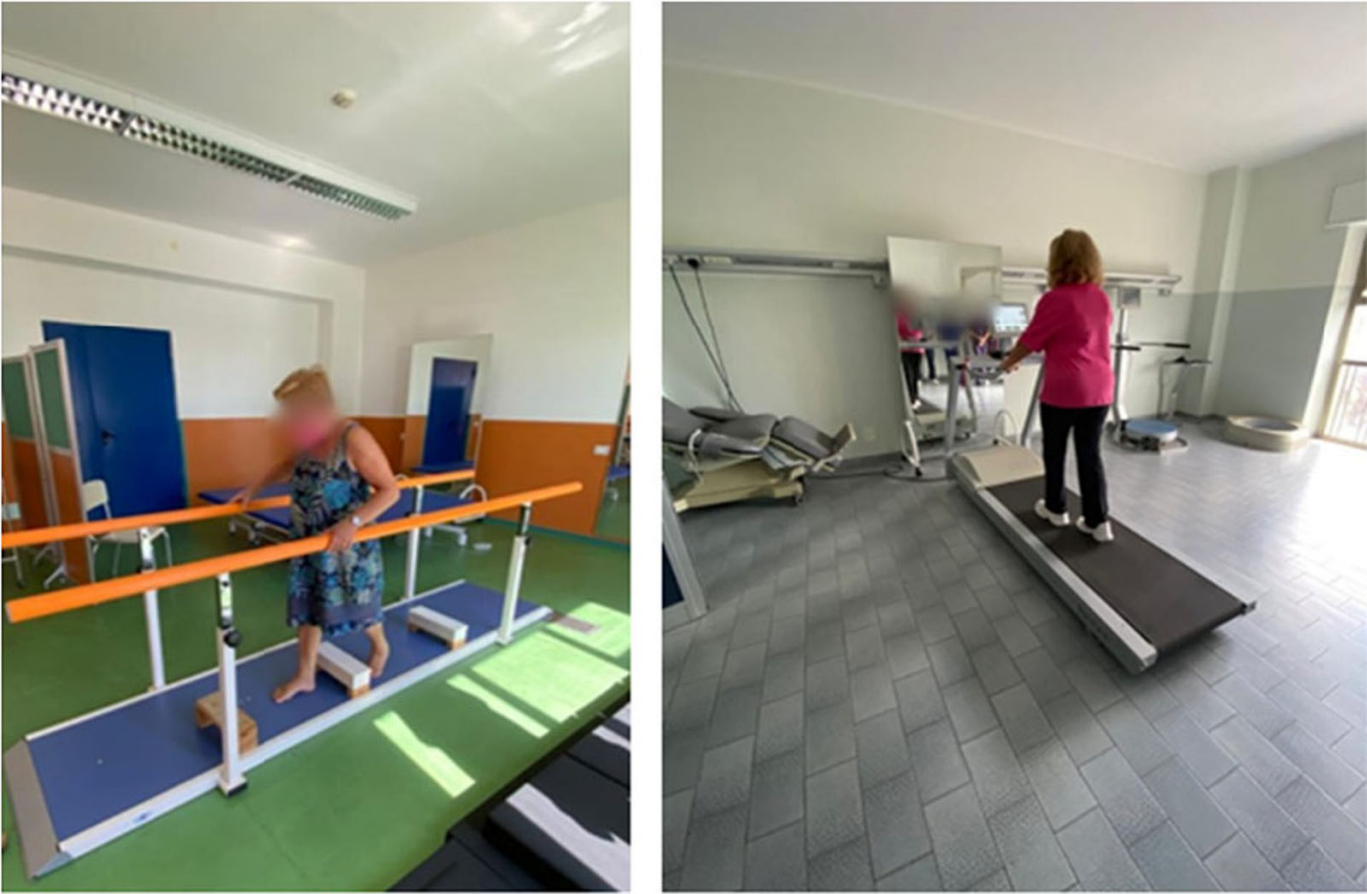
Gait training with obstacles and treadmill session.

To consolidate the learned gait pattern and enhance endurance, exercises are then performed on a treadmill system. As stability improves, patients transition from walking between parallel bars to more independent movement. Balance training, using wobble boards and coordination exercises, is crucial for improving safety and autonomy in daily activities. Visual input plays a key role in aiding anticipatory postural adjustments during these exercises also performing treadmill session (see Figure 5 for further details).

### Outcome measures

To evaluate the condition of the patients and the treatment outcomes, various physiatric and physiotherapeutic assessment scales were administered at different time‐points: T0: at baseline, before the start of infiltrative and rehabilitative treatment; T1: at the end of the treatment; T2: 3 months after the first visit; T3: 6 months after the first visit and T4: 12 months after the first visit.

Primary outcome was the HHS for the evaluation of hip functioning. This scale was developed to assess various hip‐related disabilities, focusing on four main aspects: pain, functionality, absence of deformity and joint mobility [31].

Secondary outcomes were: Pain, using the NRS [15]; balance and gait, using the Tinetti scale [39]; quality of life, using the EuroQol‐5 Dimension (EQ‐5D) [34] and physical performance, using the 6MWT [43].

### Statistical analysis

The results of the various assessments, collected and organized in a database, were analysed using the JAMOVI software (version 2.2.5). The experimental data were processed using the independent samples *t* test (or Mann–Whitney *U* for non‐parametric data) and repeated measures analysis of variance (ANOVA). Specifically, these tests were applied to all outcomes: the primary outcome, HHS; and the secondary outcomes: NRS for pain, Tinetti scale for balance and gait, EQ‐5D for quality of life and the 6MWT for physical performance. The results were expressed as means and standard deviations. These data were then presented in the form of graphs and tables according to the established outcomes. All outcomes were analysed for within‐group and between‐group comparisons across different time points. G‐Power statistics module from Jamovi software (2.4.14) was used to estimate the proper sample size on the primary outcome (HHS). We considered an alpha level of 0.05 with 80% power and a minimum effect size of 0.40. Thus, through a repeated‐measures ANOVA of group relations, a fit sample size was established to be 20 subjects per group.

## RESULTS

**Figure 6:**
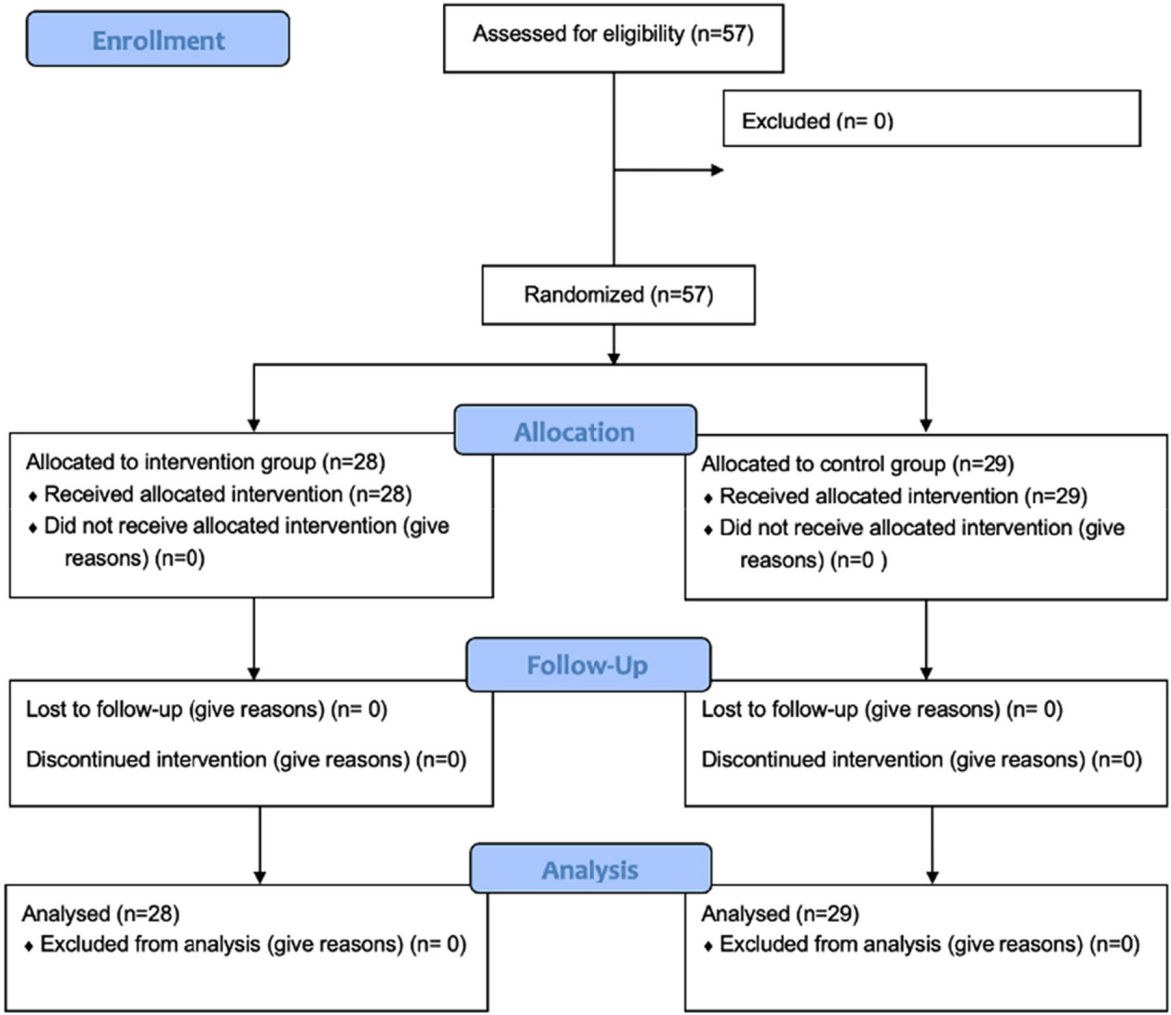
CONSORT flowchart.

A total of 71 patients were screened, of whom 57 met the inclusion criteria and were randomized and assigned to two groups, with no statistically significant differences between them at baseline, as depicted in Figure 6. All participants completed the prescribed exercise programme without discontinuation. Specifically, 28/28 in the study group and 29/29 in the control group completed all sessions.

The study group included 28 patients, while the control group consisted of 29 patients. For more details, see Table 1.

**Table 1:** Baseline characteristics of the sample.

	Study group (*n* = 28)	Control group (*n* = 29)
Males/females	5/23	7/22
Mean age (years)	66.6 ± 12.7	70.1 ± 9.56
BMI (kg/m^2^)	29.9 ± 4.68	28.67 ± 1.54

Abbreviation: BMI, body mass index.

Median baseline NRS values were 7 (interquartile range [IQR] 6–8) for the study group and 7 (IQR 6–8) for the control group. Overall, the trends in parameters show an initial improvement across nearly all parameters, followed by a stabilization phase, as depicted in Table 2.

**Table 2:** Independent samples *t* test (Mann–Whitney *U*) and repeated measures ANOVA test.

	T0 (MD ± SD)	T1 (MD ± SD)	∆T0 − T1	T2 (MD ± SD)	∆T1 − T2	T3 (MD ± SD)	∆T2 − T3	T4 (MD ± SD)	∆T3 − T4	Friedman test
			*p* value		*p* value		*p* value		*p* value	
HSS										
*p* Value test	0.601	0.239		0.404		0.205		0.465		<0.001
Study group	56.5 ± 12.3	78.4 ± 12.4	<0.001	75,6 ± 16.7	0.269	72.7 ± 15.8	0.329	73.7 ± 17.0	0.740	
Control group	54.9 ± 11.95	74.0 ± 15.20	<0.001	71.6 ± 20.02	0.615	65.5 ± 21.93	0.031	69.5 ± 8.30	0.027	
NRS										
*p* Value test	0.871	0.249		0.590		0.155		0.092		<0.001
Study group	6.71 ± 1.33	2.79 ± 2.39	<0.001	3.43 ± 2.62	0.067	3.93 ± 2.97	0.0741	3.43 ± 2.74	0.169	
Control group	6.77 ± 1.481	3.52 ± 2.50	<0.001	4.17 ± 2.568	0.762	4.89 ± 2.93	0.061	5.00 ± 1.26	0.090	
Tinetti										
*p* Value test	0.742	0.650		0.803		0.590		0.094		<0.001
Study group	22.6 ± 3.13	25.7 ± 2.77	<0.001	24.5 ± 3.76	0.089	24.6 ± 3.65	0.902	24.9 ± 3.05	0.336	
Control group	22.3 ± 5.49	25.3 ± 3.69	<0.001	26.2 ± 4.85	0.896	27.0 ± 1.00	0.076	26.6 ± 1.21	0.341	
EuroQoL										
*p* Value test	0.859	0.395		0.081		0.030		0.937		<0.001
Study group	0.578 ± 0.197	0.747 ± 0.148	<0.001	0.742 ± 0.134	0.389	0.753 ± 0.143	0.703	0.716 ± 0.192	0.116	
Control group	0.570 ± 0.171	0.709 ± 0.182	<0.001	0.637 ± 0.204	0.014	0.731 ± 0.074	0.057	0.710 ± 0.090	0.0498	
6MWT										
*p* Value test	0.619	0.399		0.581		0.742		0.114		0.378
Study group	288 ± 59.6	312 ± 90.7	0.034	282 ± 99.8	0.459	294 ± 98.4	0.496	292 ± 95.3	0.941	
Control group	277 ± 101.5	291 ± 96.5	0.111	283 ± 120.9	0.154	305 ± 150.9	0.733	345 ± 40.0	0.197	

Abbreviations: 6MWT, 6‐Minute Walking Test; ANOVA, analysis of variance; EuroQoL, European Quality of Life Scale; HHS, Harris Hip Score; MD, mean difference; NRS, Numerical Rating Scale; SD, standard deviation.

**Figure 7:**
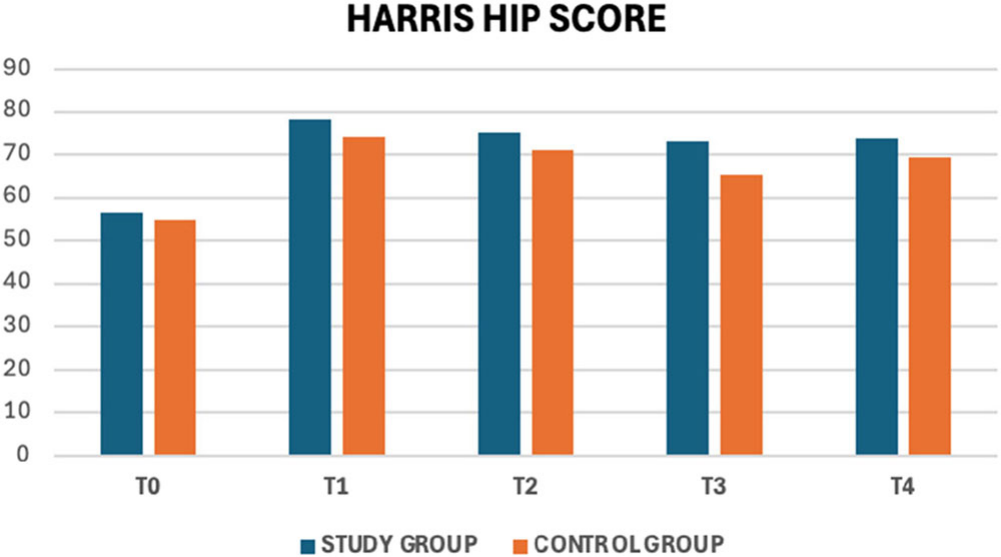
Graphical representation of the main outcome (bar chart format).

Results showed a significant improvement in hip function, particularly measured through the HHS (Figures 6 and 7).

Both groups experienced an initial increase after treatment. However, while the study group maintained stable scores over time, the control group showed a slight decline at later follow‐ups. Despite slightly better results in the study group, differences between groups were not statistically significant. Pain reduction, assessed through the NRS, followed a similar pattern. Both groups experienced a significant decrease immediately after treatment. Over time, pain levels tended to increase again, but the study group consistently reported lower scores at each follow‐up. However, statistical analysis did not confirm a significant difference between the two groups.

Other functional and quality‐of‐life measures, including balance with the Tinetti test, walking ability evaluated by 6MWT and overall well‐being assessed by EuroQoL, showed improvements in both the study and control groups.

However, when comparing outcomes between the two groups (intergroup comparisons), statistically significant differences were identified in specific secondary outcomes: while the primary outcome, the HHS, did not show statistically significant differences between the groups on direct comparison (*p* = 0.705), analysis revealed a significant advantage for the study group in measures related to mobility and balance.

Specifically, the overall analysis of the Tinetti scale, assessing balance and gait, revealed a modest but statistically significant advantage for the study group compared to the control group (*p* = 0.041). The comparison between groups for physical performance, measured by the 6MWT, demonstrated a statistically significant difference (*p* = 0.029) in favour of the combined treatment group, suggesting better functional mobility outcomes. For quality of life, a statistically significant difference between the groups emerged specifically at the 6‐month follow‐up (T3, *p* = 0.030), indicating a potentially more sustained benefit in quality of life for the group receiving HPLT.

## DISCUSSION

This is the first RCT to evaluate the efficacy of a combined treatment consisting of intra‐articular hyaluronic acid injections, HPLT and physiotherapy in patients with OA. This approach was compared to a standard treatment adopted in the control group, which consisted of hyaluronic acid injections, physiotherapy and sham HPLT.

The observed improvements were clinically relevant, surpassing the Minimal Clinically Important Difference (MCID) thresholds for the respective scores. Specifically, the MCID for the HHS is established at 7–10 points, and the MCID for the NRS is 2 points. The initial recovery observed in the study group largely exceeded these thresholds: for HHS, the increase from baseline (T0: 56.5 ± 12.3) to post‐treatment (T1: 78.4 ± 12.4) represents an improvement of approximately 21.9 points. For NRS, the reduction in pain from T0 (6.71 ± 1.33) to T1 (2.79 ± 2.39) represents a decrease of approximately 3.92 points. Furthermore, the improvements in secondary outcomes such as the 6MWT and EuroQoL also exceeded typical MCID values, confirming the substantial clinical relevance of the initial functional and quality of life gains. The main results of this RCT reported that the addition of HPLT could increase, but more importantly, maintain the initial improvements in results obtained just after the end of treatment.

Linear hyaluronic acid, known for its analgesic and anti‐inflammatory properties and its ability to stimulate endogenous synthesis by synoviocytes, seemed particularly useful in hip OA, where synovial fluid undergoes mechanical and biological alterations due to the degenerative process [9]. This finding is consistent with recent evidence highlighting the role of hyaluronic acid injections in improving pain and joint function in hip OA patients, with sustained benefits over time [40]. On the other hand, active HPLT might exploit its anti‐inflammatory, analgesic and biostimulator effects, supporting the achievement of rehabilitation goals. Nevertheless, physical therapy remains the key approach by focusing on restoring joint mobility and strengthening the stabilizing muscles of the pelvis, improving gait quality [35].

### HHS

Regarding the primary outcome of the study, HHS, the paired samples test revealed a marked improvement in the study group between T0 and T1 (56.53 ± 12.3 to 78.35 ± 12.4, *p* < 0.001), indicating significant initial recovery. However, in the subsequent phases (ΔT1–T2, ΔT2–T3 and ΔT3–T4), the changes were not significant, suggesting that after the initial improvement, the score tends to stabilize. In the control group, the statistical analysis showed a significant improvement between T0 and T1 (54.9 ± 11.95 to 74.0 ± 15.20, *p* < 0.001), indicating a substantial recovery in hip function. However, no clear improvements were observed in later phases, and in some instances, a slight decline was recorded—for example, between T2 and T3, the score decreased (71.08 ± 20.02 to 65.5 ± 21.93, *p* = 0.031), before rising again at T4 (69.55 ± 8.30). Regarding the independent samples t‐test and intergroup analysis, no statistically significant differences were observed between the two groups. However, the study group consistently showed slightly higher scores than the control group, particularly from T1 onward. The repeated measures ANOVA test confirmed a significant difference across time points (*p* < 0.001), indicating an overall improvement in hip function. However, the interaction between time and group was not significant (*p* = 0.249), suggesting similar progress in both groups. Likewise, the direct comparison between groups was not significant (*p* = 0.705), indicating that the treatment or intervention did not lead to a significant difference between the study and control groups.

Data analysis revealed greater effectiveness of the combined treatment, particularly concerning the primary outcome measured using the HHS. The maintenance of hip joint functionality in the experimental group can be attributed not only to the stimulation of endogenous hyaluronic acid synthesis and the strengthening of hip musculature but also to the biostimulatory effects of laser therapy. This result is shown by the study conducted by Vilabril et al., which demonstrated an overall superiority of HA in managing pain in patients with OA [46]. These findings are and are further supported by recent literature emphasizing the benefits of multimodal conservative management in hip OA [33].

#### Numerical Rating Scale (NRS) for Pain

Both the experimental and control groups experienced a significant reduction in pain levels from baseline (T0) to the end of treatment (T1), indicating effective short‐term pain relief. However, in the following weeks, pain levels tended to rise again, especially in the control group. At the final time point (T4), the study group maintained lower pain scores compared to the control group, although differences between the two groups were not statistically significant. Overall, pain perception improved over time in both groups (*p* < 0.001), but no significant group effect or time‐group interaction was found. These findings suggest that while both treatments were effective in reducing pain initially, the combined protocol with HPLT may have contributed to a more sustained analgesic effect. This trend aligns with previous studies, such as that by Mostafa et al., which reported beneficial effects of HPLT on pain and physical function in patients with knee osteoarthritis (KOA) [26].

### Tinetti scale

Regarding the outcome related to balance and gait improvement, assessed using the Tinetti scale, both groups showed significant improvement in balance and gait between baseline (T0) and the end of treatment (T1), with scores remaining stable in the following time points. Although no significant differences were observed between groups at individual time points, the overall analysis revealed a significant improvement over time (*p* < 0.001) and a modest advantage in the study group compared to the control (*p* = 0.041), suggesting slightly better long‐term outcomes when HPLT was included.

### 6MWT

The study group showed a significant improvement in walking distance from T0 to T1, while the control group did not demonstrate significant changes at any time point. Over time, no clear trends or interactions were observed in either group. However, a statistically significant difference between groups (*p* = 0.029) suggests that the combined treatment may have led to better overall functional mobility outcomes. These results are similar to those reported by Nazari et al., who observed significant improvements in the HILT group among individuals with OA [29].

### EuroQoL 5D

Both groups showed significant improvements in quality of life from baseline to the end of treatment (T0–T1). However, while the study group maintained stable scores over time, the control group experienced a decline between T1 and T2. A statistically significant difference between groups emerged only at T3 (*p* = 0.030), indicating a potentially greater and more sustained quality of life improvement in the study group.

The improvement in all these parameters clearly highlights the effectiveness of the rehabilitation treatment proposed in both groups. However, the results achieved in the study group compared to the control group are likely due to the significant reduction in pain symptoms, thanks to the combined action of laser therapy and intra‐articular injections. This resulted in greater physical and psychological well‐being and increased motivation to follow the physiotherapy treatment according to the clinical practice guideline for physical therapy in patients with hip or KOA [45].

We would like to emphasize that this is the first RCT that assesses the effectiveness of a combined approach that includes intra‐articular hyaluronic acid injections, HPLT and physiotherapy in enhancing hip joint function in patients with OA. A strength of our study is the adoption of a combined therapeutic approach, which, according to the literature, appears more effective than each intervention considered individually. A similar approach was shown by Ip et al., who concluded that hyaluronic acid injections together with low‐level laser therapy also prolong the longevity of degenerative joints [16]. Our results align with those reported in other studies, both in terms of the duration of laser therapy cycles and the use of physical therapy. For example, several studies have demonstrated the efficacy of laser therapy in cycles ranging from 7 to 12 sessions. Nives Stiglić‐Rogoznica et al. showed that High‐Intensity Laser Therapy (HILT) provides immediate analgesic effects in the treatment of KOA [42]. Additionally, a study led by Akaltun et al. found that in a cohort of 40 patients, combining HILT with therapeutic exercises—administered five times per week for 2 weeks—resulted in significant improvements in pain management for individuals with knee OA [1]. Although the use of laser therapy still raises questions in the rehabilitation treatment of hip OA, the benefits of intra‐articular hyaluronic acid injections and specific physiotherapy treatment are well‐documented in scientific literature [30, 36].

The results of this study should be interpreted considering some limitations. Firstly, there is a lack of long‐term follow‐up, and the number of participants should be increased to ensure greater statistical robustness and generalizability of major findings. Secondly, the lack of three‐arm allocation might limit certain conclusions, as a potential additional control group without hyaluronic acid injection. Thirdly, regarding the follow‐up, unfortunately, we were not able to consider whether the two types of approach prolonged the timing of a potential total hip replacement.

However, to the best of our knowledge, this is the first study that assessed the efficacy of a combination of intra‐articular hyaluronic acid injections, HPLT and physiotherapy in improving hip joint function in hip OA patients.

## CONCLUSIONS

Taken together, findings of this RCT highlighted how a combined therapeutic approach, consisting of ultrasound‐guided intra‐articular injections of a hyaluronic acid‐based hydrogel, integrated with physiotherapy and active HPLT, might be effective in achieving clinically significant improvements in hip function and pain reduction in patients with hip OA.

Furthermore, the inclusion of HPLT showed statistically significant advantages in specific secondary outcomes, notably the 6‐MWT and Quality of Life at 6 months, suggesting a better sustained functional mobility compared to the control group. Although this RCT confirmed the short‐term and mid‐term clinical benefits, the current scientific literature regarding HPLT in hip OA remains limited. Therefore, continuing RCTs with extended follow‐up periods is essential to fully confirm the long‐term effectiveness of this combined therapy and to investigate whether this approach can prolong the time until a potential total hip replacement is required.

## AUTHOR CONTRIBUTIONS

*Conceptualization*: Alessandro de Sire. *Methodology*: Alessandro de Sire, Umile Giuseppe Longo and Antonio Ammendolia. *Formal analysis*: Nicola Marotta and Emanuele Prestifilippo. *Investigation*: Alessandro de Sire, Isabella Bartalotta, Roberta Zito and Federica Pisani. *Resources*: Antonio Ammendolia. *Data curation*: Nicola Marotta, Isabella Bartalotta, Emanuele Prestifilippo and Roberta Zito. *Writing—original draft*: Alessandro de Sire, Nicola Marotta, Isabella Bartalotta and Emanuele Prestifilippo. *Writing—review and editing*: Francesco Agostini, Umile Giuseppe Longo and Antonio Ammendolia. *Visualization*: Roberta Zito, Valerio Ammendolia and Federica Pisani. *Supervision*: Alessandro de Sire, Umile Giuseppe Longo and Antonio Ammendolia. All authors have read and agreed to the published version of the manuscript.

## FUNDING INFORMATION

The authors have no funding to report.

## CONFLICT OF INTEREST STATEMENT

The authors declare no conflict of interest.

## ETHICS STATEMENT

This study was approved by the Regional Ethical Committee with approval number 429/2021 (date of approval: 16 December 2021). All the patients signed and approved the informed consent.

## Data Availability

The dataset is available from the corresponding author on reasonable request.
